# Mitochondrial RNA as a broad-spectrum DAMP coordinates antiviral and damage-related immune clearance and confers therapeutic potential

**DOI:** 10.1038/s41419-026-09153-7

**Published:** 2026-08-04

**Authors:** Mingfu Tian, Guangli Li, Zelin Chai, Siyu Liu, Zhiqiang Li, Dehua Zhang, Chengliang Zhu, Kailang Wu, Xinghui Liu

**Affiliations:** 1 State Key Laboratory of Virology and Biosafety, College of Life Sciences, Wuhan University, Wuhan, China; 2 Postgraduate Training Base at Shanghai Gongli Hospital, Ningxia Medical University, Shanghai, China; 3 Department of Clinical Laboratory, Renmin Hospital of Wuhan University, Wuhan, China; 4 School of Gongli Hospital Medical Technology, University of Shanghai for Science and Technology, Shanghai, China; 5 Department of Clinical Laboratory, Pudong Gongli Hospital, Shanghai University of Medicine & Health Sciences, Shanghai, China

**Keywords:** Autoimmunity, Immune evasion

## Abstract

During viral infection and tissue injury, efficient immune clearance of infected or damaged cells is crucial for host defense and homeostasis. Here, we identify mitochondrial RNA (mtRNA) as a broad-spectrum damage-associated molecular pattern (DAMP) that coordinates antiviral and damage-related immune clearance. Through an integrative approach combining in vitro cellular assays and multi-strain murine models, we demonstrate that viral infection and cellular stress promote POLRMT-dependent mtRNA synthesis and its release into the cytosol through BAX/mPTP-mediated mitochondrial pores. Once released, mtRNA activates the MAVS signaling pathway, triggering a robust type I interferon response that operates independently of the cGAS-STING axis. Notably, viruses exploit a negative feedback loop for immune evasion: type I interferon upregulates the exoribonuclease PNPT1, which degrades cytosolic mtRNA and thereby dampens the mtRNA-MAVS axis. Critically, pharmacological inhibition of PNPT1 with lanthanum chloride (LanC), combined with BH3 mimetics that relieve the BCL-2-mediated blockade of BAX/BAK pores, synergistically reactivates mtRNA release and restores antiviral immunity. This dual strategy demonstrates potent antiviral and anti-fibrotic efficacy in preclinical models without significant toxicity. Our findings establish the mtRNA-MAVS axis as a central, broadly applicable immune surveillance pathway and provide a mechanistic framework for developing therapies that overcome both viral immune evasion and the limitations of current STING-targeted agonists.

## Introduction

Within the biological context of pathogen invasion and cellular stress, the host maintains internal homeostasis through a precisely regulated immune surveillance system [1–3]. To breach host defense barriers, viruses have evolved multi-layered immune evasion strategies [4–7]: Firstly, the formation of double-membrane viral replication organelles (VROs) that physically encapsulate viral nucleic acids within isolated vesicles, thereby evading recognition of pathogen-associated molecular patterns (PAMPs) by pattern recognition receptors (PRRs) [8–10]. Secondly, epigenetic modifications of viral nucleic acids—such as 5’-triphosphate group shielding or RNA methylation—that specifically disrupt conformational activation of PRRs like RIG-I [11–15]. These mechanisms significantly reduce the efficiency of viral nucleic acid recognition by the host innate immune system, compelling the organism to initiate compensatory signaling pathways to restore immune surveillance. Notably, under oxidative stress or genotoxic pressure, damage-associated molecular patterns (DAMPs), despite lacking canonical viral nucleic acid features, activate immune responses analogous to pathogen infection through yet unelucidated mechanisms. This phenomenon suggests the potential involvement of conserved endogenous danger signals across diverse stress types, which may mediate cross-talk between pathogen-induced and stress-induced immune activation [16, 17].

As central hubs for cellular energy metabolism and key regulators of innate immunity [18–21], mitochondria release mitochondrial DNA (mtDNA) that potently activates immune responses. This property has been extensively validated as a critical danger signal in stress contexts such as cellular damage and radio/chemotherapy [22–27]. While mtDNA functions as an endogenous immunostimulatory signal, its immune-activating effects strictly depend on the cGAS-STING signaling pathway. However, this pathway exhibits significant inherent limitations: Its expression displays high tissue specificity, manifesting as epigenetic silencing particularly in terminally differentiated cells (e.g., neurons, myocytes) and specialized anatomical regions (e.g., renal medulla, hepatic lobules) [28–32]. Furthermore, multiple pathogen-associated molecular patterns and damage signals—including viral infections—fail to effectively activate this pathway [30, 33]. More critically, current agonist therapies targeting cGAS-STING have encountered developmental setbacks due to insufficient clinical response rates. Notably, a subset of cells does not express STING [28, 34], yet the tissues comprising these cells are precisely common sites for viral infection, tumorigenesis, and malignant transformation. Consequently, as cells are persistently exposed to microenvironmental stressors that threaten homeostasis, they must maintain a finely tuned balance between pro-survival and pro-death signaling pathways to preserve tissue integrity. This homeostatic mechanism strongly suggests the existence of a broader, more universal signaling pathway that helps maintain immune equilibrium. The prevailing dual dilemma—characterized by both “expression-blind” and “stimulus-blind” landscapes—compounded by translational failures, further underscores the urgent need to establish broadly effective immune pathways. This is particularly critical for developing compensatory mechanisms within STING-unresponsive tissues to achieve a breakthrough.

In this study, we identify mitochondrial RNA (mtRNA) as a broad-spectrum DAMP that coordinates antiviral and damage-related immune clearance. We demonstrate that viral infection and cellular stress promote POLRMT-dependent mtRNA synthesis and its release into the cytosol through BAX/mPTP-dependent mitochondrial pores. Once released, mtRNA activates the MAVS signaling pathway, triggering a potent innate immune response. Critically, MAVS is widely expressed across diverse tissues, ensuring pan-tissue efficacy of this pathway.

We further reveal that viruses exploit a negative feedback mechanism for immune evasion: type I interferon (IFN-β) induces the expression of polynucleotide phosphorylase 1 (PNPT1), a 3’ -5’ exoribonuclease that degrades cytosolic mtRNA, thereby dampening the mtRNA-MAVS axis. Targeting this vulnerability, we show that pharmacological inhibition of PNPT1 (lanthanum chloride, LanC) combined with BH3 mimetics—which remove the BAX/BAK blockade by suppressing anti-apoptotic BCL-2 proteins—synergistically reactivates mtRNA release and restores antiviral immunity. This dual strategy demonstrates potent antiviral and anti-fibrotic efficacy in preclinical models without significant toxicity.

Our findings establish the mtRNA-MAVS axis as a central, broadly applicable immune surveillance pathway and provide a mechanistic framework for developing therapies that overcome both viral immune evasion and the limitations of current STING-targeted agonists.

## Results

### mtRNA as a broad-spectrum DAMP triggering immune responses to infection and stress

**Fig. 1:**
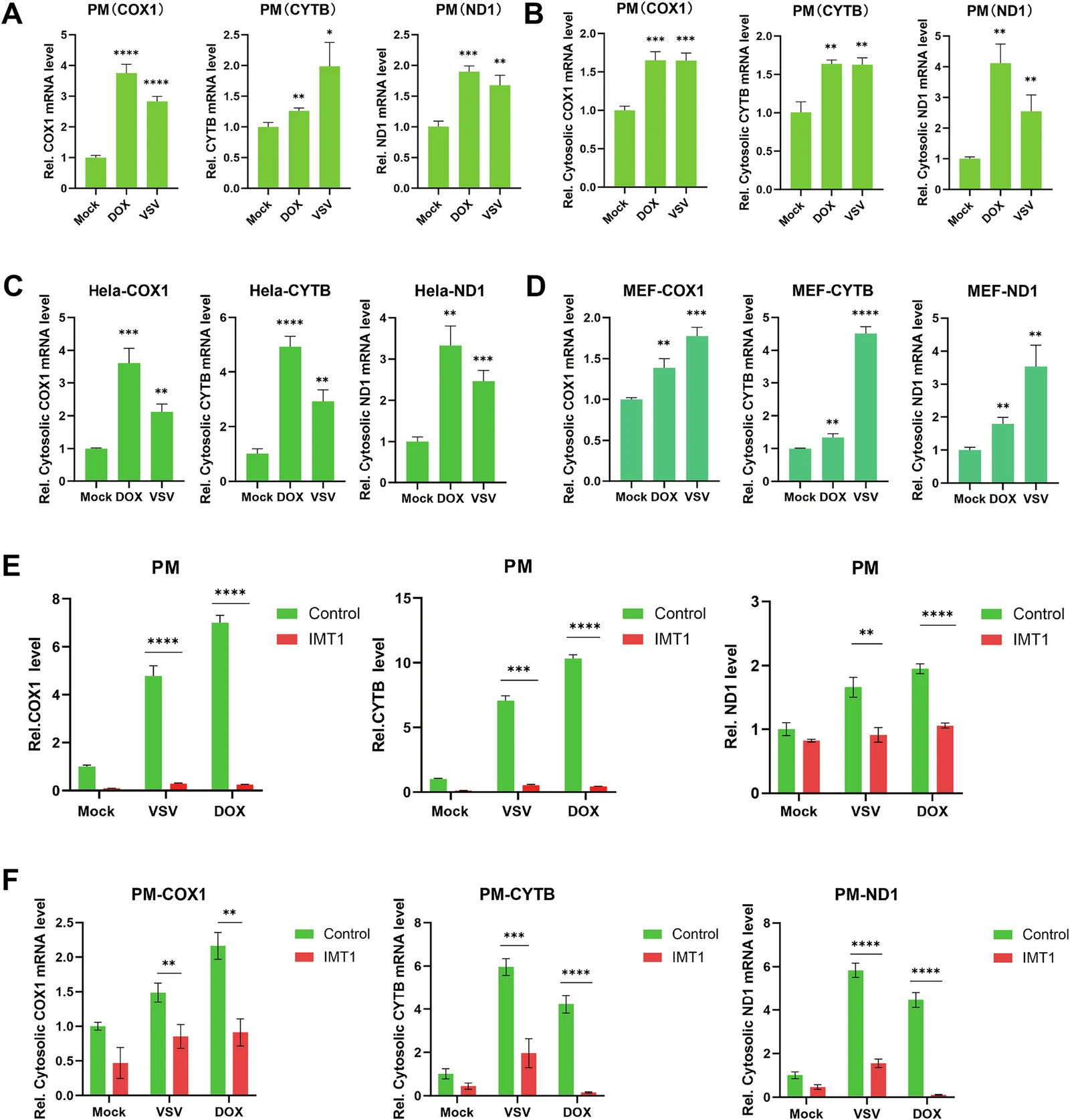
Mechanisms of virus- and stress-induced cytosolic mtRNA release. **A** PM cells were treated with DOX (4 h) and VSV (4 h). Total mtRNA levels were assessed by qRT-PCR. **B** PM cells were treated with DOX (4 h) and VSV (4 h). Cytosolic mtRNA levels were analyzed by qRT-PCR. **C** HeLa cells were treated with DOX (4 h) or VSV (4 h). Cytosolic mtRNA levels were analyzed by qRT-PCR. **D** MEF cells were treated with DOX (4 h) or VSV (4 h). Cytosolic mtRNA levels were analyzed by qRT-PCR. **E** PM cells were pretreated with the mitochondrial RNA polymerase inhibitor IMT1 (2 h), followed by stimulation with DOX or VSV (4 h). Total mtRNA levels were assessed by qRT-PCR. **F** PM cells were pretreated with the mitochondrial RNA polymerase inhibitor IMT1 (2 h), followed by stimulation with DOX or VSV (4 h). Cytosolic mtRNA levels were analyzed by qRT-PCR. Data are presented as mean ± SEM and are representative of at least two independent experiments. Statistical significance was determined by Student’s *t* test; *P < 0.05, **P < 0.01, ***P < 0.001, ****P < 0.0001.

During the long-term evolutionary interplay between pathogens and the host, viruses have evolved strategies to evade host pattern recognition receptors (PRRs) [35, 36]. This may compel the host to rely on endogenous compensatory signaling pathways to sustain antiviral defenses. Additionally, under non-infectious stress, cells can activate similar innate immune responses via damage-associated molecular patterns (DAMPs), suggesting the existence of conserved endogenous signaling mechanisms. To test the central hypothesis that mitochondrial RNA (mtRNA) acts as such a damage-associated molecular pattern (DAMP), we first examined the levels and localization of mtRNA in mouse primary macrophages (PMs) following stimulation with a model genotoxic drug, doxorubicin (DOX), and a model virus, vesicular stomatitis virus (VSV). Relative to mock-treated controls, both DOX and VSV significantly upregulated the expression of mitochondrial genome-encoded transcripts—COX1, CYTB, and ND1 (Fig. 1A). Subcellular fractionation and qRT-PCR revealed that this increase was also reflected in the cytosolic compartment, with a marked release of mtRNA into the cytosol under both stress and infection conditions (Fig. 1B–D).

We next investigated the drivers of this mtRNA accumulation and release. Consistent with previous reports linking oxidative stress to mitochondrial damage [37–40], we ruled out reactive oxygen species (ROS) as a primary driver, as ROS scavenging with N-acetylcysteine (NAC) did not reduce mtRNA levels (Fig. S1A). Similarly, while NF-κB signaling was activated, its inhibition (BAY11-7082) blocked downstream IL-6 but did not affect mtRNA levels (Fig. S1B), confirming that mtRNA accumulation is an upstream stress response, not a consequence of inflammatory signaling. These results point to a regulated cellular process rather than a passive byproduct.

Given that mitochondrial transcription is mediated by the mitochondrial RNA polymerase (POLRMT), we evaluated its role. Treatment of host cells with the POLRMT-specific inhibitor IMT1 robustly suppressed the stress- and infection-induced elevation of both total and cytosolic mtRNA levels (Fig. 1E, F). These findings demonstrate that diverse stressors, including genotoxic damage and viral infection, enhance POLRMT-dependent mtRNA synthesis and its subsequent release into the cytosol. This mechanism, conserved across different stimuli, likely serves as a foundational step for innate immune activation via endogenous RNA sensing.

### Viral infection and cellular stress trigger immunity via mtRNA release through mitochondrial pores

After establishing that diverse stressors enhance POLRMT-dependent mtRNA synthesis and release, we next investigated the mechanism by which mtRNA exits the mitochondria to enter the cytosol.

**Fig. 2:**
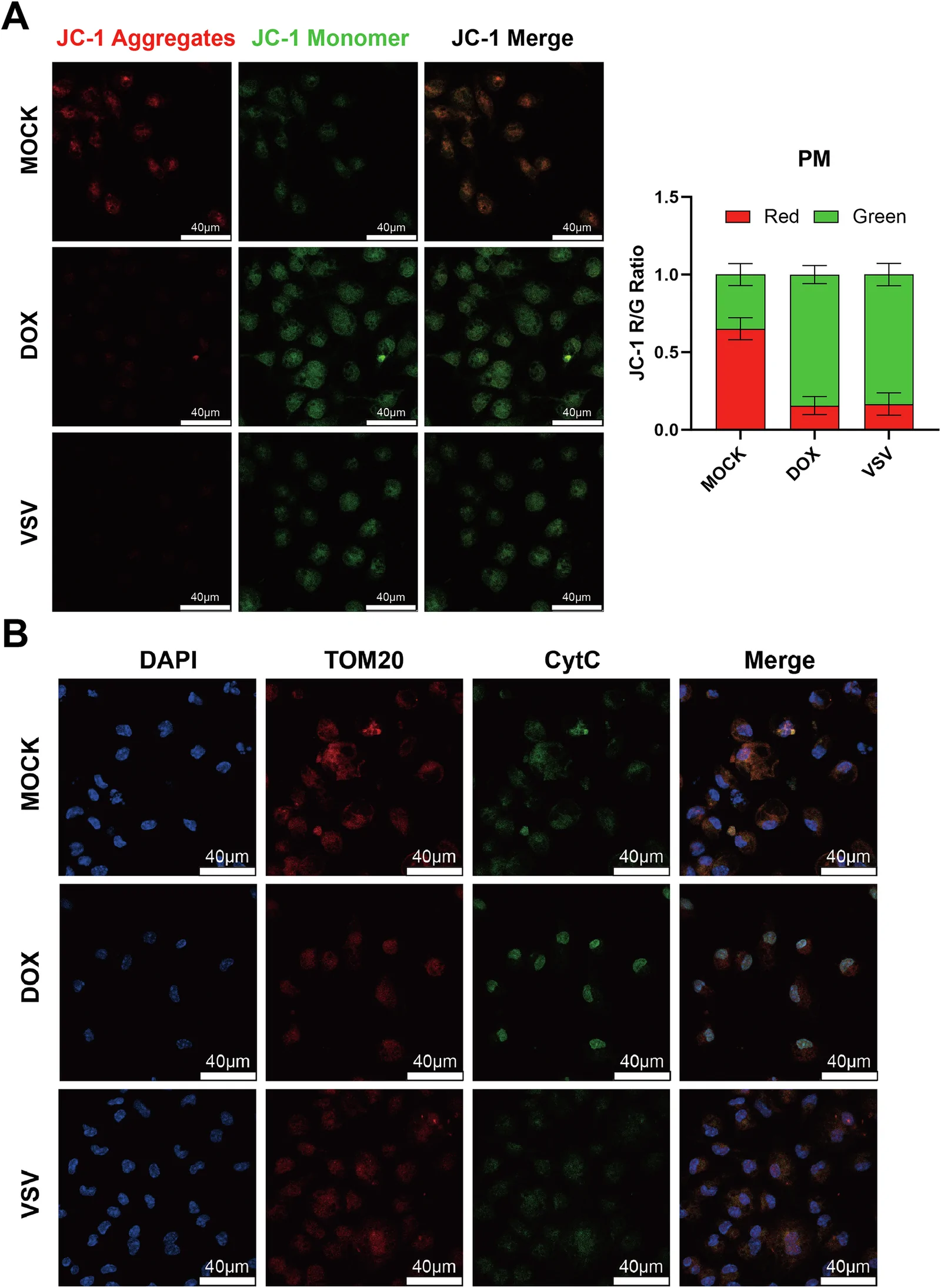
Mechanisms of virus- and stress-induced mitochondrial damage and mtRNA release. **A** Mitochondrial membrane potential (ΔΨm) was assessed using JC-1 staining. PM cells were treated with Mock (control), DOX (4 h), or VSV (4 h). JC-1 aggregates represent high membrane potential (red fluorescence), while monomers represent low membrane potential (green fluorescence). The bar graph on the right quantifies the JC-1 red/green (R/G) ratio (Red: red aggregates; Green: green monomers). **B** Immunofluorescence analysis of mitochondrial permeability transition and cytochrome c (CytC) release. PM cells were treated with Mock, DOX, or VSV. Cells were stained with DAPI (blue, nucleus), TOM20 (red, mitochondrial outer membrane protein), and CytC (green, cytochrome c). Merge images illustrate mitochondrial morphology and the translocation of CytC from mitochondria to the cytosol.

We first assessed changes in mitochondrial status by measuring mitochondrial membrane potential (ΔΨm) and the release of the pro-apoptotic protein cytochrome C (CytC). Using JC-1 staining, we observed that both DOX and VSV treatment in PM cells led to a significant depolarization of the mitochondrial membrane, as evidenced by a decrease in the JC-1 Red/Green fluorescence ratio (Fig. 2A). This functional change was corroborated by immunofluorescence imaging, which showed a marked release of CytC from the mitochondria into the cytosol under both stress conditions, accompanied by a decrease in the colocalization between CytC and the mitochondrial outer membrane marker TOM20, indicating increased mitochondrial permeability (Fig. 2B).

**Fig. 3:**
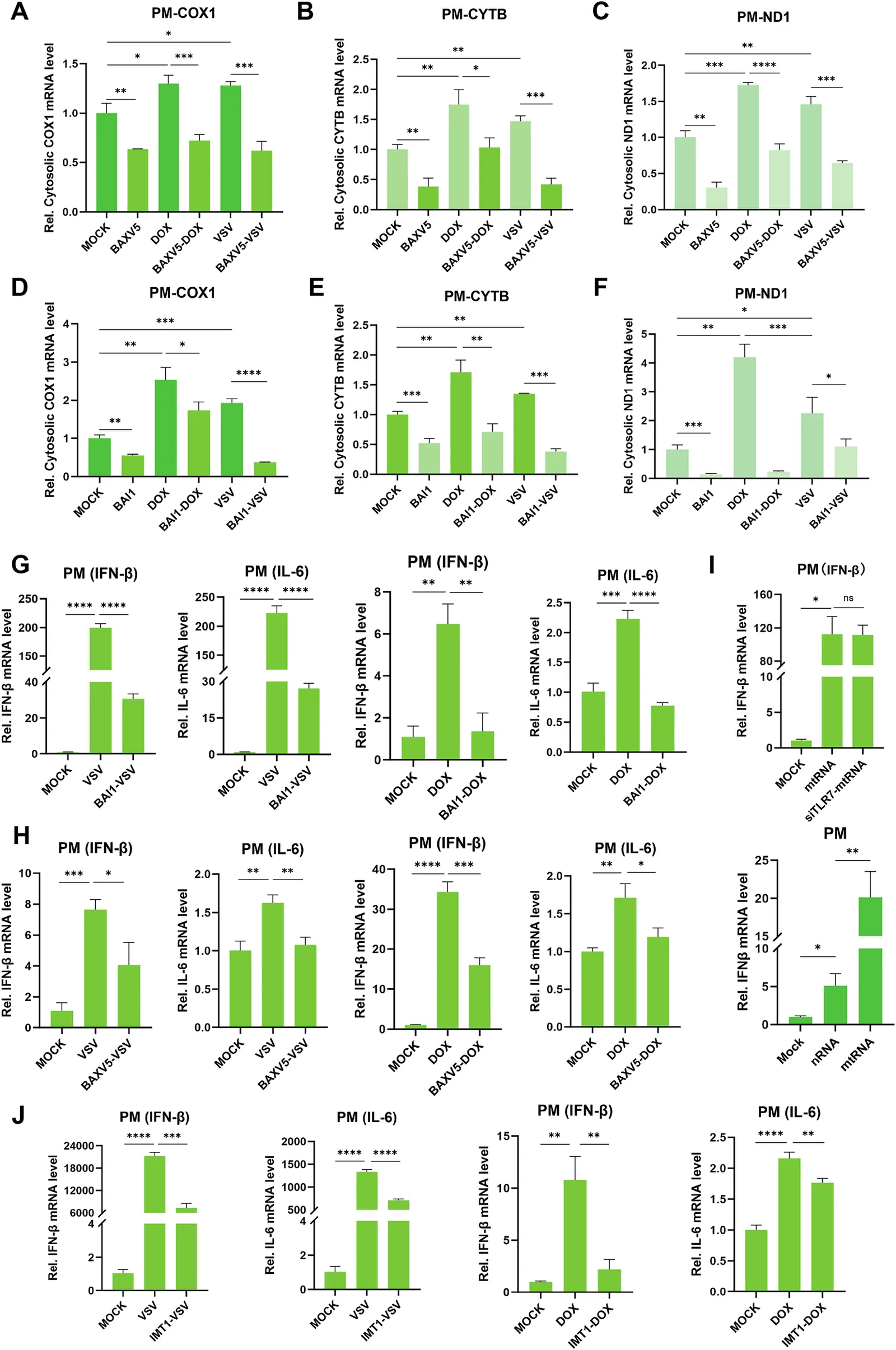
BAI1 and BAXV5 regulate cytosolic mtRNA release and antiviral cytokine production in PM cells under viral or stress stimulation. **A–C** PM cells were pretreated with BAXV5 (2 h) followed by stimulation with DOX or VSV (4 h). Cytosolic mtRNA levels (COX1, CYTB, ND1) were analyzed by qRT-PCR. **D–F** PM cells were pretreated with BAI1 (2 h) followed by stimulation with DOX or VSV (4 h). Cytosolic mtRNA levels (COX1, CYTB, ND1) were quantified by qRT-PCR. **G, H** PM cells were pretreated with BAI1 or BAXV5 (2 h) followed by stimulation with DOX or VSV (4 h). IFN-β and IL-6 mRNA levels were assessed by qRT-PCR. **I** Relative IFN-β mRNA levels were evaluated by qRT-PCR in PM cells treated with mtRNA (12 h), siTLR7-mtRNA, nRNA, or siTLR7-nRNA (for the latter two groups, cells were pretreated with siRNA for 24 h prior to nucleic acid administration for 12 h). **J** PM cells were pretreated with BAI1 (2 h) followed by stimulation with DOX or VSV (4 h). IFN-β and IL-6 mRNA levels were measured by qRT-PCR. Data are presented as mean ± SEM and are representative of at least two independent experiments. Statistical significance was determined by Student’s *t* test; *P < 0.05, **P < 0.01, ***P < 0.001, ****P < 0.0001.

These results demonstrate that viral infection and genotoxic stress induce mitochondrial dysfunction and promote the opening of mitochondrial pores. We hypothesized that this permeabilization provides the physical route for mtRNA release. To test this, we quantified the levels of mitochondrial-encoded transcripts (COX1, CYTB, ND1) in the cytosolic fraction of PM cells. Consistent with the observed mitochondrial damage, both DOX and VSV stimulation significantly increased the levels of all three mtRNA species in the cytosol (Fig. 3A–F). This increase was potently blocked by pre-treatment with BAXV5, an inhibitor of the BAX pore, and BAI1, an inhibitor of the mitochondrial permeability transition pore (mPTP) (Fig. 3A–F). These findings indicate that mitochondrial permeabilization via BAX-dependent MOMP and mPTP is a prerequisite for stress-induced mtRNA release into the cytosol.

To further test whether this mtRNA release directly drives innate immune activation, we measured downstream cytokine production. PM cells were pretreated with BAI1 (Fig. 3G) or BAXV5 (Fig. 3H) and then stimulated with DOX or VSV. Both inhibitors significantly suppressed the mRNA levels of IFN-β and IL-6, demonstrating that blocking mtRNA release attenuates the subsequent immune response.

To independently confirm the immunostimulatory capacity of mtRNA, we transfected purified mtRNA from donor cells into naïve recipient PMs. This transfection significantly upregulated IFN-β and IL-6 mRNA levels in a manner independent of the classical TLR7 pathway (Fig. 3I). Notably, mtRNA exhibited a stronger immunostimulatory capacity compared to an equivalent amount of nuclear-derived RNA (Fig. 3I).

Finally, to establish the crucial role of POLRMT-dependent mtRNA synthesis in this pathway, we pretreated PM cells with the POLRMT inhibitor IMT1 before viral infection. IMT1 significantly suppressed VSV- and DOX-induced upregulation of IFN-β and IL-6 in PM cells (Fig. 3J). This confirms that POLRMT-mediated mtRNA production is a critical rate-limiting step for viral activation of innate immunity.

Collectively, these data demonstrate that diverse stressors induce mitochondrial membrane permeabilization, leading to the POLRMT-dependent synthesis and subsequent BAX/mPTP-dependent release of mtRNA into the cytosol. This released mtRNA then acts as a potent DAMP to trigger a robust immune response.

### The POLRMT-mtRNA axis drives host innate immune responses

Having demonstrated that POLRMT-dependent mtRNA synthesis is essential for stress-induced innate immune activation at the cellular level, we next sought to validate the physiological relevance of this axis in vivo using a murine model.

**Fig. 4:**
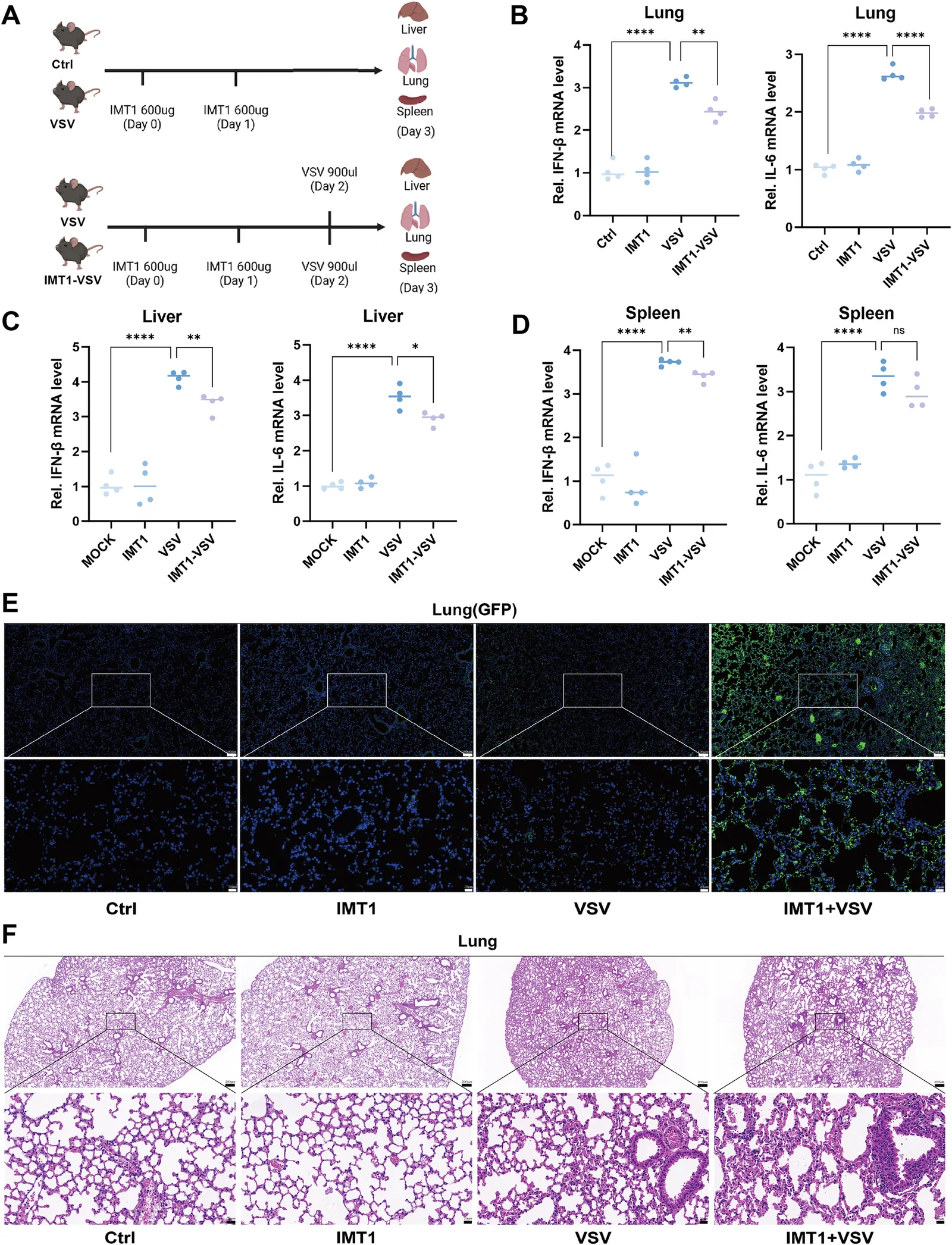
Mitochondrial stress regulates virus-induced innate immunity in vivo. **A** Experimental timeline. C57BL/6 (*n* = 4) and MAVS⁻/⁻ (*n* = 4) mice received intraperitoneal (i.p.) injections of IMT1 (600 μg/kg per dose) on Days 0 and 1, followed by i.p. challenge with vesicular stomatitis virus (VSV, 900 μl) on Day 2. Lung, liver, and spleen tissues were harvested on Day 3 for downstream analyses. Relative mRNA levels of IFN-β and IL-6 in the lung (**B**), liver (**C**), and spleen (**D**) were quantified by qRT-PCR. **E** Immunofluorescence staining for GFP in lung tissues. **F** H&E staining of lung tissues. Data are presented as mean ± SEM and are representative of at least two independent experiments. Statistical significance was determined by Student’s *t* test; *P < 0.05, **P < 0.01, ***P < 0.001, ****P < 0.0001.

C57BL/6 mice were randomly assigned to four groups: vehicle control (Ctrl); IMT1 alone; VSV infection alone; and IMT1 pretreatment followed by VSV infection (IMT1 + VSV). Mice received intraperitoneal injections of IMT1 (600 µg/kg per dose) on Days 0 and 1 to suppress POLRMT-mediated mtRNA biosynthesis, followed by intraperitoneal inoculation with VSV on Day 2. Animals were euthanized 24 h post-viral exposure (Day 3), and liver, lung, and spleen tissues were systematically collected for multimodal analyses (Fig. 4A).

Quantitative RT-PCR analysis revealed that VSV infection robustly upregulated IFN-β and IL-6 mRNA levels in lung, liver, and spleen tissues compared to vehicle controls (Fig. 4B–D). Notably, pre-treatment with IMT1 significantly altered this immune response landscape. In lung tissues, the IMT1 + VSV group exhibited a marked reduction in IFN-β and IL-6 mRNA levels compared to the VSV-only group (Fig. 4B). Similar inhibitory trends were observed in the liver and spleen, confirming that POLRMT-mediated mtRNA synthesis is a critical upstream regulator of the virus-induced innate immune response across multiple organs (Fig. 4C, D).

To further assess the functional consequences of POLRMT inhibition, we evaluated viral replication by quantifying GFP reporter gene expression. IMT1 pre-treatment synergistically enhanced VSV-GFP fluorescence in the lung, liver, and spleen compared to VSV infection alone (Figs. 4E and S2A–E). These results indicate that suppressing mtRNA synthesis impairs the host’s antiviral immune response, thereby creating a permissive environment for enhanced viral replication.

Histopathological examination via H&E staining showed that IMT1 intervention alone did not induce additional tissue damage compared to vehicle controls, as tissue architecture remained intact (Figs. 4F and S2F, G). However, in the VSV infection group, IMT1 pretreatment exacerbated tissue pathology, particularly in the lungs, where alveolar structure disruption was more pronounced compared to the VSV-only group (Fig. 4F). This is consistent with the observed increase in viral replication.

Collectively, these in vivo findings provide compelling evidence that the POLRMT-mtRNA axis is a pivotal regulator of antiviral innate immunity. Inhibiting this axis not only attenuates the host’s cytokine response but also enhances viral dissemination and associated tissue pathology, highlighting POLRMT as a central molecular mechanism coupling mitochondrial stress with antiviral immune signaling.

### mtRNA activates innate immune responses via the MAVS pathway

Having demonstrated that stress-induced mtRNA release triggers innate immune activation, we next investigated the upstream sensor mediating this effect. Given the central role of the mitochondrial antiviral signaling protein (MAVS) pathway in RNA sensing, we hypothesized that MAVS mediates the mtRNA-driven immune cascade.

To test this, we first performed a loss-of-function analysis in MAVS knockout (MAVS-/-) cells. VSV infection robustly induced IFN-β mRNA expression in wild-type (WT) mouse embryonic fibroblasts (MEFs), whereas this response was completely abolished in MAVS-/- MEFs (Fig. S3A), confirming MAVS as an essential mediator of antiviral immunity. More directly, we transfected purified mtRNA from donor peritoneal macrophages (PMs) into recipient PMs. As shown in Fig. S3B, C, mtRNA significantly upregulated IFN-β and IL-6 mRNA expression in WT PMs, but this effect was entirely absent in MAVS-/- PMs. These results provide the first direct evidence that mtRNA activates macrophage immune responses via a MAVS-dependent pathway.

To investigate the physiological relevance of this mtRNA-MAVS axis in clearing damaged or stressed cells, we employed an oncogenic transformation model using hydrodynamic injection of myr-AKT and NRASV12 into WT and MAVS-/- mice. Immunohistochemical analysis at Day 12 post-injection revealed that the number of CD3⁺ T cells was significantly higher in WT liver tissues compared to MAVS-/- counterparts, while N-RAS protein expression and activation were markedly enhanced in MAVS-deficient livers (Fig. S3D). These findings suggest that MAVS deficiency impairs innate immune surveillance and pre-cancerous cell clearance. Long-term monitoring indicated that visible tumor nodules developed in the liver of MAVS-/- mice after 5 weeks, whereas no tumors were observed in WT mice (Fig. S3D). These results demonstrate that the mtRNA-MAVS axis is essential for immune surveillance against oncogenic stress.

Collectively, these data establish mtRNA as a DAMP that activates the MAVS pathway, a mechanism critical for both antiviral immunity and the elimination of stressed/transformed cells to maintain tissue homeostasis.

### IFN-β-mediated PNPT1 upregulation attenuates innate immune activation

**Fig. 5:**
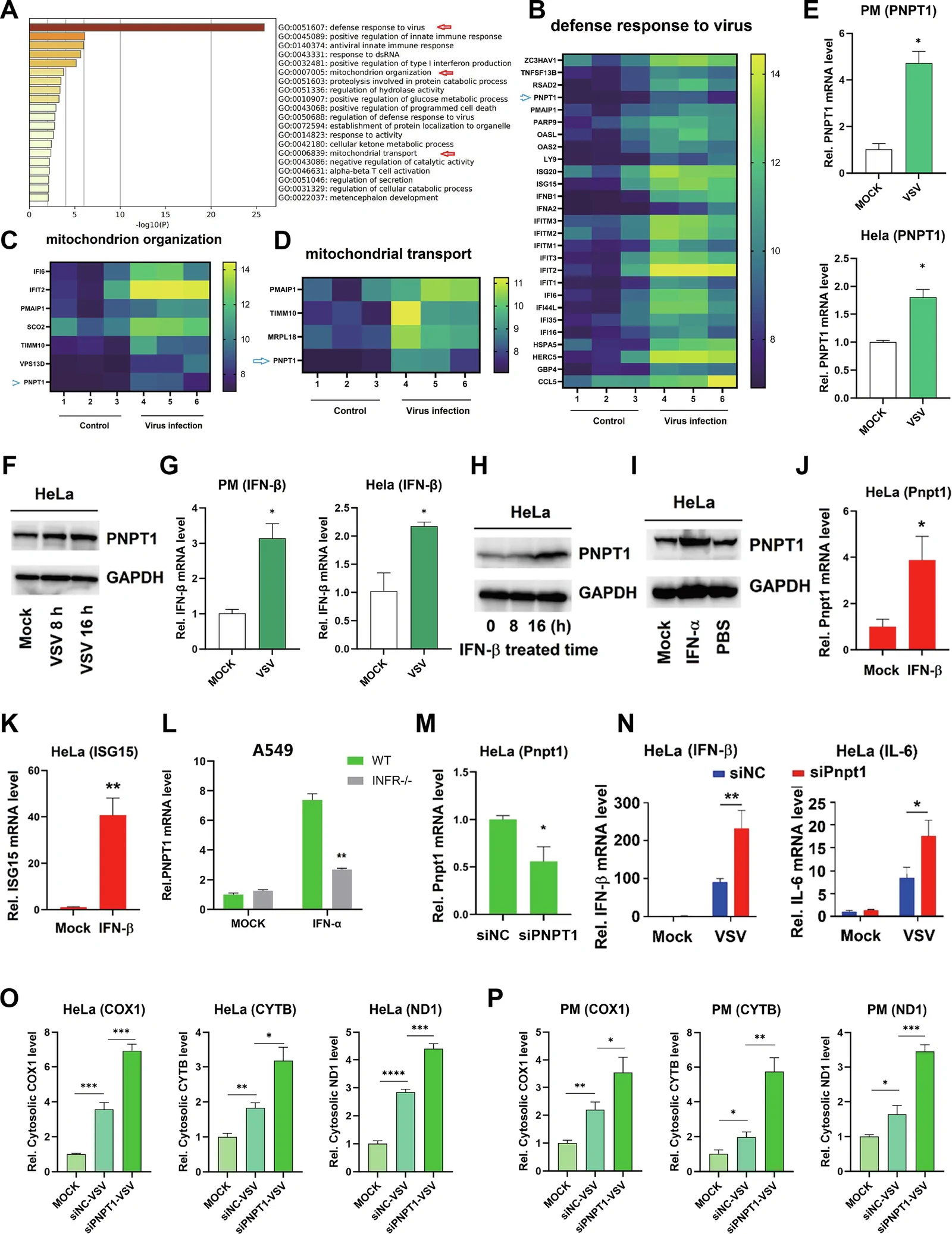
PNPT1 functions as a negative regulator of mitochondrial RNA-mediated antiviral immunity. **A** Gene Ontology (GO) enrichment analysis of upregulated genes in control versus IAV-infected monocytes from the GSE35473 dataset. **B–D** Heatmaps showing gene expression patterns across pathways identified in (**A**). **E** PNPT1 mRNA levels in mouse peritoneal macrophages (PMs) infected with VSV (4 h) were measured by qRT-PCR. **F** PNPT1 protein levels in HeLa cells infected with VSV (8 h or 16 h) were analyzed by Western blot (WB). **G** IFN-β mRNA levels in mouse peritoneal macrophages (PMs) infected with VSV (4 h) were measured by qRT-PCR. PNPT1 protein levels in HeLa cells treated with recombinant IFN-β (**H**) or IFN-α (**I**) were assessed by WB. **J, K** PNPT1 and ISG15 mRNA levels in HeLa cells treated with recombinant IFN-β (8 h or 16 h) were measured by qRT-PCR. **L** PNPT1 mRNA levels in WT and IFNAR-/- A549 cells treated with recombinant IFN-α (16 h) were quantified by qRT-PCR. **M** HeLa cells were infected with siPnpt1 or siNC for 48 h. PNPT1 mRNA levels were analyzed by qRT-PCR. **N** HeLa cells infected with siPNPT1 or siNC (48 h) were challenged with VSV (12 h). IFN-β and IL-6 mRNA levels were measured by qRT-PCR. **O** Cytosolic mtRNA levels were quantified by qRT-PCR in HeLa cells transfected with siPNPT1 or siNC (48 h) followed by VSV challenge (12 h). **P** Cytosolic mtRNA levels were quantified by qRT-PCR in PMs transfected with siPNPT1 or siNC (48 h) followed by VSV challenge (12 h). Data are presented as mean ± SEM and are representative of at least two independent experiments. Statistical significance was determined by Student’s *t* test; *P < 0.05, **P < 0.01, ***P < 0.001, ****P < 0.0001.

Since mtRNA-MAVS signaling is a critical host defense mechanism, we reasoned that viruses would evolve strategies to subvert it. To identify factors negatively regulating this pathway, we analyzed the gene expression dataset GSE35473 from monocytes post-viral infection. Gene Ontology (GO) enrichment analysis revealed that upregulated pathways upon infection were predominantly associated with antiviral immunity, mitochondrial organization, and mitochondrial transport (Fig. 5A). Intersection analysis of these gene sets identified polynucleotide phosphorylase 1 (PNPT1) as a common hub gene (Fig. 5B–D), pointing to a potential role in regulating mitochondrial nucleic acid metabolism and immune responses.

We next asked how viral infection might drive PNPT1 expression. VSV infection significantly upregulated PNPT1 mRNA in both PMs and HeLa cells, and this was accompanied by a clear increase in PNPT1 protein levels in HeLa cells (Fig. 5E, F). Similar induction by VSV and HSV was confirmed in MLFs and PMs (Fig. S4A, B). These results indicate that viruses can actively induce PNPT1 expression during infection.

Since VSV is known to activate the mtRNA-MAVS pathway and trigger type I interferon production, we hypothesized that IFN-β could be the key intermediate linking viral infection to PNPT1 upregulation. Consistent with this idea, VSV potently induced IFN-β mRNA expression in both HeLa cells and PMs (Fig. 5G). More directly, treating HeLa cells with recombinant IFN-β alone was sufficient to elevate PNPT1 protein and mRNA levels, along with ISG15 mRNA (Fig. 5H, J, K). Recombinant IFN-α had a similar effect on PNPT1 protein expression (Fig. 5I). This induction was entirely dependent on the type I interferon receptor, as the upregulation of PNPT1 mRNA by IFN-α was completely abolished in IFNAR-KO A549 cells compared to wild-type controls (Fig. 5L). These results establish an “Virus → IFN-β/IFNAR → PNPT1” negative feedback loop, suggesting that viruses can exploit this loop to dampen the early innate immune response.

We next assessed the functional impact of this virus-induced PNPT1 expression. siRNA-mediated silencing of PNPT1 (siPNPT1) in HeLa cells significantly enhanced VSV-induced IFN-β and IL-6 mRNA expression (Fig. 5M, N). Similarly, siPNPT1 boosted IAV- and H₂O₂-induced IFN-β expression in primary macrophages (PMs) (Fig. S4C, D). To determine whether this enhanced immune response was driven by increased availability of cytosolic mtRNA, we directly measured mtRNA levels upon PNPT1 knockdown. We found that siPNPT1 induced increased mtRNA release across multiple cell types (Fig. S4E–H). Notably, PNPT1 silencing markedly elevated the cytosolic levels of mitochondrial-encoded transcripts (COX1, CYTB, ND1) in both HeLa cells and PMs following VSV infection (Fig. 5O, P).

Together, these results establish that PNPT1 is a key negative regulator induced by type I interferon, which enables viral immune evasion by reducing cytosolic mtRNA levels. This “IFN-β-PNPT1-mtRNA” feedback loop provides a mechanistic basis for viral immune evasion and identifies PNPT1 as a compelling target for therapeutic intervention.

### Pharmacological inhibition of PNPT1 synergizes with BH3 mimetic drugs, unlocking dual antiviral and anti-fibrotic therapy via mitochondrial RNA release

Previous studies have demonstrated that mitochondrial RNA (mtRNA) can effectively activate antiviral immunity, while host cells counteract this pathway by upregulating PNPT1 protein, thereby facilitating viral immune evasion. Given the persistent challenges in the clinical translation of STING agonists and the current lack of therapies targeting the mtRNA–MAVS signaling axis, this study pioneers a novel therapeutic strategy aimed at the PNPT1–mtRNA axis. We focused on lanthanum chloride (LanC), an FDA-approved drug known to inhibit PNPT1, though its antiviral efficacy has not been systematically validated. The limited efficacy of PNPT1 monotherapy is attributed to tissue-specific overexpression of anti-apoptotic BCL-2 family members (BCL-2, BCL-xL, MCL-1), which suppress mtRNA release by blocking BAK/BAX pore formation. To address this limitation, we developed a combinatorial system comprising LanC and BH3 mimetics (navitoclax, venetoclax, targeting BCL-2/BCL-xL/BCL-w), designed to dually antagonize PNPT1 and BCL-2 family proteins, thereby robustly activating the BAK/BAX-mediated apoptotic cascade and promoting mtRNA release.

Experimental results indicated that although administration of LanC or BH3 mimetics alone was capable of activating an immune response, the combination treatment significantly enhanced the magnitude of the immune activation (Fig. S5A). Moreover, BTSA1—a specific activator of BAK channel formation—also markedly induced immune responses (Extended Data Fig. S5B). This study further demonstrated that the drug-induced immune activation depends on the POLRMT-mediated synthesis and release of mtRNA (Fig. S5C–E). To evaluate the safety profile of the combination regimen, we systematically assessed the acute toxicity of co-administration of BH3 mimetics and LanC in healthy C57BL/6 mouse models. Results showed that the combination treatment caused only mild body weight fluctuation throughout the course of therapy, with recovery to control levels by the later phase (Fig. S5F). Histopathological examination revealed no significant damage in major organs, confirming the safety of the drug combination (Fig. S5G–J).

To further evaluate the protective effect of the combination strategy in tissues with low STING expression, we utilized a cisplatin (CIPT)-induced mouse model of acute injury and compared the effects of IMT1 monotherapy and the combination of LanC and ABT263. The results showed that in lung tissue, H&E staining indicated aggravated injury after IMT1 treatment, whereas the combination of LanC and ABT263 effectively alleviated pathological damage (Fig. S5M). Furthermore, TUNEL staining revealed increased apoptosis in the IMT1 group, which was significantly reduced in the combination therapy group (Fig. S5N), demonstrating that this treatment regimen provides a tissue-protective effect in a STING-deficient context.

**Fig. 6:**
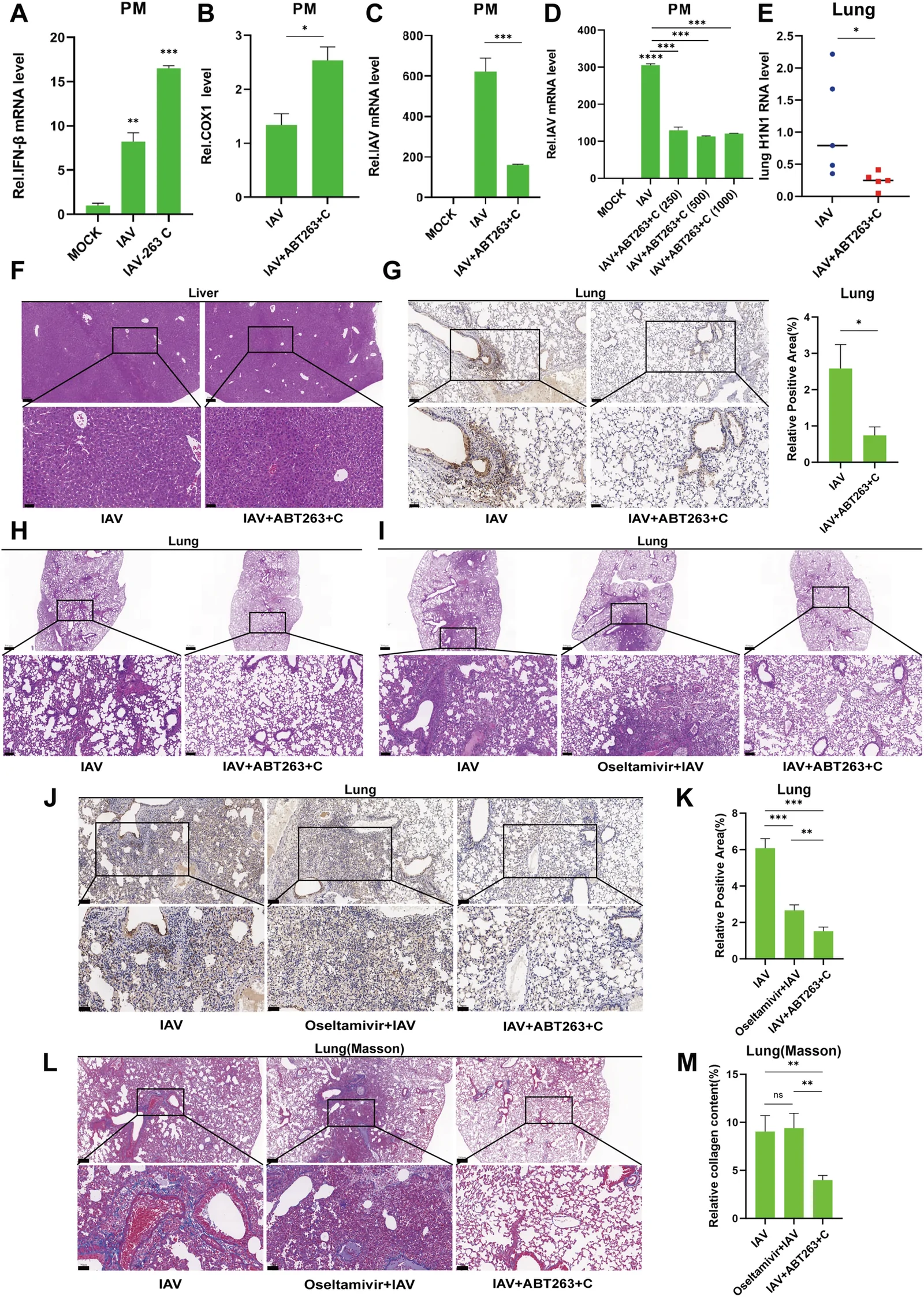
Combined therapy enhances antiviral efficacy and reduces lung fibrosis in IAV-infected mice. IFN-β (**A**), COX1 (**B**), and IAV mRNA (**C**) levels in PM cells infected with IAV or co-treated with IAV + ABT-263 + Lanatoside C were measured by qRT-PCR. **D** IAV mRNA levels in PM cells infected with IAV alone or co-treated with IAV + ABT-263 + Lanatoside C were quantified by qRT-PCR. **E** C57BL/6 mice infected with IAV and treated with ABT-263 + Lanatoside C for 7 days. Lung H1N1 RNA levels were analyzed by qRT-PCR. H&E staining of liver (**F**) and lung (**H, I**) tissues. **G, J, K** Viral nucleoprotein (NP) staining and quantitative analysis in lung tissues. Masson’s trichrome staining (**L**) and quantitative assessment (**M**) of pulmonary fibrosis in lung tissues. Data are presented as mean ± SEM and are representative of at least two independent experiments. Statistical significance was determined by Student’s t test; *P < 0.05, **P < 0.01, ***P < 0.001, ****P < 0.0001.

Finally, the therapeutic potential of this combination strategy was evaluated in the context of antiviral treatment. In the model of influenza A virus (IAV)-infected peritoneal macrophages (PMs), combined treatment with ABT-263 and LanC significantly upregulated the expression of COX1 (Fig. 6B) and IFN-β (Fig. 6A). This therapeutic approach reduced IAV mRNA levels (Fig. 6C) in a manner that was not obviously dose-dependent across the tested range (Fig. 6D). To validate these effects in vivo, an IAV-infected C57BL/6 J mouse model was established. In lung tissues, combination therapy markedly reduced H1N1 RNA load (Fig. 6E). Histopathological analysis revealed that the combination treatment of ABT-263 and LanC did not induce significant hepatotoxicity (Fig. 6F) and markedly alleviated IAV-induced lung tissue injury (Fig. 6H). Immunohistochemical (IHC) analysis further demonstrated a significant reduction in the viral nucleoprotein (NP)-positive area in the ABT-263 plus LanC combination treatment group (Fig. 6G). A similar trend was observed in the ABT-199 plus LanC combination group, where histopathological evaluation indicated that lung tissue damage was substantially mitigated, and immunofluorescence staining showed a pronounced decrease in NP-positive areas (Fig. S5K, L). During the 7-day post-infection treatment period, the ABT-263 + LanC combination was more potent against the virus than oseltamivir (Fig. 6I). Although both oseltamivir monotherapy and ABT-263 + LanC combination treatment inhibited NP expression, the combination therapy exhibited a stronger suppressive effect (Fig. 6J, K). Masson’s trichrome staining further demonstrated that ABT-263 + LanC combination treatment alleviated lung fibrosis in IAV-infected mice, an effect not observed with oseltamivir treatment (Fig. 6L, M). These findings indicate that the combination of BH3 mimetics with LanC can effectively counteract viral replication and associated tissue damage.

Our findings highlight multiple advantages of the combination therapy strategy. By synergistically targeting both the PNPT1-mediated immunosuppressive pathway (via LanC) and the anti-apoptotic BCL-2 family proteins (via BH3 mimetics), the combined treatment effectively removes two major barriers to mtRNA release. It is noteworthy that in tissues with low STING expression, as demonstrated in the CIPT-induced acute injury model, the combination of LanC and ABT263 exhibited significant tissue-protective effects, markedly attenuating pathological damage and apoptosis. This indicates the potential of this strategy even under conditions of cGAS-STING signaling deficiency. In both in vitro and IAV-infected models, the ABT-263/LanC combination regimen demonstrated a favorable safety profile. The precise immunological mechanisms underlying the observed antiviral and anti-fibrotic effects, including the involvement of specific inflammatory pathways, require further investigation. Nonetheless, these results establish a foundation for novel antiviral therapies targeting the mitochondrial-immune axis, offering a mechanistically supported strategy to overcome the current clinical limitations of STING agonists and address drug-resistant infections.

## Discussion

The innate immune system is critical for both pathogen defense and tissue homeostasis. It operates by sensing external pathogen-associated molecular patterns (PAMPs) and internal damage-associated molecular patterns (DAMPs). In this work, we identify mitochondrial RNA (mtRNA) as a ubiquitous and potent DAMP that bridges the gap between cellular stress, viral infection, and innate immune activation.

A clear picture emerges from our data. First, diverse stressors—from genotoxic drugs to viral infections—drive POLRMT-dependent mtRNA synthesis. This newly synthesized mtRNA then exits the mitochondria through BAX/mPTP pores. Once in the cytosol, it is sensed by the MAVS pathway, which in turn triggers a robust type I interferon response. This cascade is remarkably consistent: it operates across genotoxic stress (DOX), viral infection (VSV, IAV), and in multiple cell types, underscoring its broad physiological relevance. Viruses, however, have found a way to exploit this very system. Our data reveal an elegant immune evasion strategy centered on the IFN-β-PNPT1 negative feedback loop. The same interferon response that fights the virus also upregulates the exoribonuclease PNPT1. PNPT1, in turn, degrades cytosolic mtRNA, effectively shutting down the host’s own alarm signal. This makes PNPT1 a kind of “checkpoint” in the mtRNA-MAVS axis.

This understanding leads directly to a therapeutic opportunity. We found that simply inhibiting PNPT1 with LanC is not enough, because the BCL-2 family of anti-apoptotic proteins keeps the BAX/BAK pores closed, preventing new mtRNA from being released. A dual strategy is required. By combining LanC with BH3 mimetics (which remove the BCL-2 brake), we effectively relieve both roadblocks. This unlocks a powerful antiviral response and, notably, also provides anti-fibrotic benefits. This two-pronged approach is not just about killing the virus; it’s about repairing the damage.

Importantly, this entire pathway operates independently of the cGAS-STING axis. Given that STING expression is highly tissue-specific and that STING agonists have faced clinical setbacks, the mtRNA-MAVS pathway—with its broad tissue distribution—offers a compelling alternative for therapeutic development. The favorable safety profile we observed in our preclinical models further supports the feasibility of this strategy.

Some questions remain. A more detailed characterization of the immune microenvironment—such as measuring cytokine levels in serum and bronchoalveolar lavage fluid, or tracking myeloid cell recruitment to the lungs—is needed and will be the focus of future work. The long-term efficacy and safety of this combinatorial approach in chronic models also warrants further exploration. Overall, our work defines the mtRNA-MAVS axis as a central, broadly expressed immune surveillance mechanism. It provides a clear mechanistic framework for designing a new class of antiviral and anti-fibrotic therapies that can overcome both viral immune evasion and current clinical limitations of STING-targeted drugs.

## Materials and methods

All animal procedures were approved by the Institutional Animal Care and Use Committee of Wuhan University and conducted in accordance with relevant guidelines (Animal Use Protocol #SKLV-AE2024-029). Viral infection (IAV, VSV) and disease models were established in C57BL/6 mice, followed by pharmacological interventions and tissue collection for molecular and histological analyses. Cell culture experiments utilized various mammalian cell lines treated with chemical compounds or infected with viruses, with transfection techniques employed for genetic manipulations. Standard molecular biology methods including immunoblotting, qRT-PCR, ELISA, and mitochondrial/cytosolic RNA isolation were performed, with statistical analysis conducted using Student’s t-test.

### Animal study

Wild-type C57BL/6 mice were purchased from the Hubei Provincial Center for Laboratory Animal Research (Wuhan, China). MAVS-knockout mice on the C57BL/6 background were kindly provided by Dr. Hongbin Shu (Wuhan University). All animal experimental protocols were reviewed and approved by the Institutional Animal Care and Use Committee (IACUC) of the College of Life Sciences at Wuhan University, and procedures were conducted in strict accordance with the U.S. Animal Welfare Act and the National Institutes of Health (NIH) Guidelines for the Care and Use of Laboratory Animals in Biomedical Research.

Establishment of IAV Infection Model and Drug Treatment: Six- to eight-week-old C57BL/6 mice were intranasally inoculated with IAV to establish a viral infection model. Beginning on day 2 post-infection, mice were divided into the following treatment groups and administered compounds via intraperitoneal injection for three consecutive days: ABT199 + LanC combination group (20 mg/kg); ABT263 + LanC combination group (20 mg/kg); Oseltamivir monotherapy group (20 mg/kg, i.p.). At 24 h after the final administration, euthanasia was performed under anesthesia. Tissue samples were collected for immunohistochemical staining and quantitative real-time PCR (qRT-PCR) analysis.

Establishment of VSV Infection Model and Pharmacological Intervention**:** Six- to eight-week-old C57BL/6 mice were randomly assigned to four groups (n = 4 per group): vehicle control (Ctrl), IMT1 alone, VSV alone, and IMT1 + VSV combination groups. Mice were administered IMT1 (600 μg/kg) via intraperitoneal injection for two consecutive days. Twenty-four h after the final administration, mice in the infection groups were challenged with VSV suspension (900 μL, i.p.) to establish the infection model. Tissue samples (liver, lung, and spleen) were collected 24 h post-infection for subsequent H&E staining, qRT-PCR, and viral GFP imaging analysis.

Establishment of Cancer Model and Sample Analysis : A mouse model of hepatocellular carcinoma was established using hydrodynamic injection. The Sleeping Beauty transposon system was employed to co-deliver the following plasmids for gene overexpression: pT3-myr-AKT-HA (10 µg), pT/Caggs-NRASV12 (10 µg), and pCMV (CAT)T7-SB100 (2 µg). Liver tissues were collected at 6–12 days post-injection for immunohistochemical analysis to evaluate early immune cell infiltration and N-Ras expression levels. Additional liver samples were harvested at 5–8 weeks after injection for further immunohistochemical and quantitative assessments of hepatocellular carcinoma progression.

For immunohistochemical experiments, Mouse organ tissues were fixed in 4% paraformaldehyde (24 h), paraffin-embedded, sectioned, and antigen-retrieved. Sections were blocked with 5% BSA/PBS, then stained with H&E or primary antibodies (4 °C, overnight), followed by HRP-conjugated secondary antibodies (1 h, room temperature) and DAB development. After hematoxylin counterstaining, ethanol dehydration, and resin mounting, images were captured by optical microscopy and analyzed for positive signals using ImageJ.

### Cell culture

HeLa cells, A549 cells, and B16 cells were purchased from the China Center for Type Culture Collection (CCTCC). Mouse embryonic fibroblasts (MEFs) and mouse lung fibroblasts (MLFs) were isolated from C57BL/6 wild-type or MAVS-knockout mice as previously described. Primary peritoneal macrophages (PMs) were isolated from C57BL/6 mice by thioglycolate elicitation. All cells were cultured in Dulbecco’s Modified Eagle Medium (DMEM, Gibco) supplemented with 10% fetal bovine serum (FBS, Gibco), 100 U/mL penicillin, and 100 µg/mL streptomycin. Cells were maintained at 37 °C in a humidified incubator with 5% CO₂.

### Reagents and antibodies

The following chemical reagents were used in this study: doxorubicin (DOX, TargetMol), N-acetylcysteine (NAC, TargetMol), BAY11-7082 (TargetMol), IMT1 (TargetMol), BAI1 (TargetMol), BAXV5 (TargetMol), lanthanum chloride (LanC, TargetMol), Navitoclax (ABT-263, TargetMol), Venetoclax (ABT-199, TargetMol), BTSA1 (TargetMol), and oseltamivir (TargetMol). Recombinant mouse IFN-β and IFN-α were purchased from R&D Systems.

Primary antibodies used included: rabbit anti-PNPT1 (14487-1-AP, Proteintech, 1:1000), mouse anti-GAPDH (G9295, Sigma, 1:5000), mouse anti-Cytochrome C (66264-1-Ig, Proteintech, 1:500), rabbit anti-TOM20 (11802-1-AP, Proteintech, 1:500), rabbit anti-influenza A nucleoprotein (NP, ab128193, Abcam, 1:1000), anti-F4/80 (ab111101, Abcam, 1:200), anti-CD3 (ab16669, Abcam, 1:200), and anti-N-RAS (sc-519, Santa Cruz, 1:200). Corresponding HRP-conjugated or FITC/CY3-conjugated secondary antibodies (Abbkine, 1:2000) were used as appropriate. All antibodies were diluted in 5% BSA in PBS or TBST according to the manufacturer’s instructions.

### Cell treatment

For drug treatment experiments, cells at approximately 60–70% confluency were treated with the following compounds for 6–48 h: doxorubicin (0–5 μM), hydrogen peroxide (0–100 μM), IMT1 (0–20 μM), BAXV5 (0–20 μM), BAI1 (0–20 μM), or BTSA1 (0–20 μM). Cells were harvested after treatment for subsequent analysis.

For viral infection assays, cells at a similar confluency (60–70%) were infected with vesicular stomatitis virus (VSV), herpes simplex virus (HSV), or influenza A virus (IAV). After 6–24 h of infection, cells were collected for further analysis.

### JC-1 Mitochondrial Membrane Potential Assay

PM cells were seeded on glass coverslips and divided into Mock control, DOX-treated, and VSV-infected groups for 6–12 h. Mitochondrial membrane potential (ΔΨm) was assessed using a JC-1 assay kit. Briefly, cells were incubated with JC-1 dye (5 µg/mL) at 37 °C for 20 min, washed with staining buffer, and counterstained with DAPI. Images were acquired via confocal microscopy, where red fluorescence indicates JC-1 aggregates (high ΔΨm) and green fluorescence indicates JC-1 monomers (low ΔΨm). The red/green fluorescence ratio was quantified using ImageJ to evaluate ΔΨm depolarization.

### TOM20 and cytochrome C (CytC) Co-localization assay

PM cells cultured on coverslips were treated with DOX or infected with VSV for 6–12 h, with untreated cells serving as the Mock control. Cells were fixed with 4% paraformaldehyde for 15 min, permeabilized with 0.1% Triton X-100, and blocked with 5% BSA. Samples were incubated overnight at 4 °C with primary antibodies against TOM20 (rabbit, Proteintech, 1:500) and Cytochrome C (mouse, Proteintech, 1:500). After washing, cells were incubated with corresponding Alexa Fluor-conjugated secondary antibodies (anti-rabbit 594 and anti-mouse 488) for 1 hour at room temperature, followed by DAPI nuclear counterstaining. Images were acquired using a confocal microscope.

### Transfection experiments

Transfection was performed using the liposomal reagent Lipofectamine 2000 (Thermo Fisher Scientific) to deliver the following molecules: non-specific negative control siRNA (siNC), siRNA targeting PNPT1 (siPNPT1), mitochondrial RNA (mtRNA), and siRNA targeting TLR7 (siTLR7). After transfection, cells were cultured for 12–48 h before subsequent experimental procedures.

### Immunoblotting

Cells were lysed in RIPA buffer (50 mM Tris-HCl [pH 7.5], 150 mM NaCl, 0.5 mM EDTA, 1% NP-40, 1% SDS) supplemented with protease and phosphatase inhibitors (Roche). Lysates were sonicated (3 × 10 s pulses), rotated at 4 °C for 1 h, and centrifuged at 12,000 × *g* for 15 min. Supernatants were mixed with 5× SDS loading buffer, denatured at 100 °C for 5 min, and separated by SDS-PAGE. Proteins were transferred to PVDF membranes, blocked with 5% non-fat milk, and probed with primary antibodies overnight at 4 °C. Horseradish peroxidase (HRP)-conjugated secondary antibodies were applied for 1 h at room temperature. Signals were detected using enhanced chemiluminescence (ECL).

### qRT-PCR

Total RNA was extracted using TRIzol reagent (Invitrogen) and reverse-transcribed into cDNA with HiScript III RT Mix (Vazyme Biotech, China). qRT-PCR was performed using ChamQ SYBR Master Mix (Vazyme Biotech) and gene-specific primers (Table S1) on a QuantStudio 5 system (Applied Biosystems).

### Mitochondrial RNA isolation and transfection

Mitochondria were isolated using a Mitochondrial Isolation Kit (Thermo Scientific, 89874). Total mitochondrial RNA (mtRNA) was extracted with TRIzol, quantified, and 2–5 µg mtRNA was transfected into naive cells using Lipofectamine 2000. After 12-48 h, cellular RNA was extracted for downstream analysis.

### Cytosolic RNA extraction and treatment

Cytosolic RNA was isolated using digitonin (20 μg/mL, 10 min). Cytosolic fraction purity was confirmed by immunoblotting for cytoplasmic (GAPDH) and mitochondrial markers. For cytosolic RNA transfection, 2–5 µg RNA was delivered into cells via Lipofectamine 2000. RNA was harvested after 12-48 h for qRT-PCR.

### GEO data and online data analysis

This study utilized the gene expression profile dataset (GSE35473, sourced from the GEO database) of monocytes post-viral infection. RNA sequencing (RNA-Seq) data were preprocessed through normalization and batch effect correction. Differentially expressed genes (DEGs) were screened with thresholds of |log2FC | ≥1 and FDR < 0.05. Gene Ontology (GO) enrichment analysis using the DAVID tool revealed significantly enriched pathways in the virus-infected group, including “antiviral immunity,” “mitochondrial organization,” and “mitochondrial transport.” Further integrative analysis combined gene sets related to antiviral defense and mitochondrial function. Venn diagram intersection analysis and STRING protein-protein interaction (PPI) network topological assessment (via the Cytoscape CytoHubba plugin) identified polynucleotide phosphorylase 1 (PNPT1) as a cross-pathway hub gene regulating mitochondrial nucleic acid metabolism and immune responses. All analyses were performed using R language (v4.1.2) and Bioconductor packages, with data visualization implemented via ggplot2 and ComplexHeatmap. Statistical significance was set at P < 0.05 (Benjamini-Hochberg correction for multiple testing).

### Statistical analysis

The related results were expressed as mean ± s.e.m. (standard error of the mean). Student’s *t* test of analysis between different groups was used to determine statistical significance. The Prism v9 software was used for statistical analysis. *P* value < 0.05 was statistically significant.

## Supplementary information

Supporting Information

original data

original data

Supplementary Table s1

## Data Availability

All data required to support the conclusions of this study are included in the main text and/or Supplementary Materials.
