# Supplementary material for: Mitochondrial RNA as a broad-spectrum DAMP coordinates antiviral and damage-related immune clearance and confers therapeutic potential
Source: Cell Death Dis. 2026 Aug 4;17(1):928. doi: 10.1038/s41419-026-09153-7 (PMC13724680; doi:10.1038/s41419-026-09153-7)
Supplement: Supplementary file 1 [file 41419_2026_9153_MOESM1_ESM.pdf]

Supporting Information

Mitochondrial RNA as a Broad-Spectrum DAMP Coordinates Antiviral and Damage-Related Immune Clearance and Confers Therapeutic Potential

Mingfu Tian[1#], Guangli Li[2#], Zelin Chai[1#], Siyu Liu[1], Zhiqiang Li[4], Dehua Zhang[3], Chengliang Zhu[4], Kailang Wu[1], Xinghui Liu[5]*

[1]State Key Laboratory of Virology, College of Life Sciences, Wuhan University, Wuhan 430072, China.

[2]Postgraduate training base at Shanghai Gongli hospital, Ningxia medical university, Shanghai 200135, China.

[3]School of Gongli Hospital Medical Technology, University of Shanghai for Science and Technology, Shanghai 200093, China.

[4]Department of Clinical Laboratory, Renmin Hospital, Wuhan University, Wuhan 430060, China.

[5] Department of Clinical Laboratory, Pudong Gongli Hospital, Shanghai University of Medicine & Health Sciences, Shanghai, China

[#]These authors contributed equally to this work.

***Corresponding author**. Xinghui Liu   sylhuxh@163.com

This PDF file includes:

Figs. S1 to S5

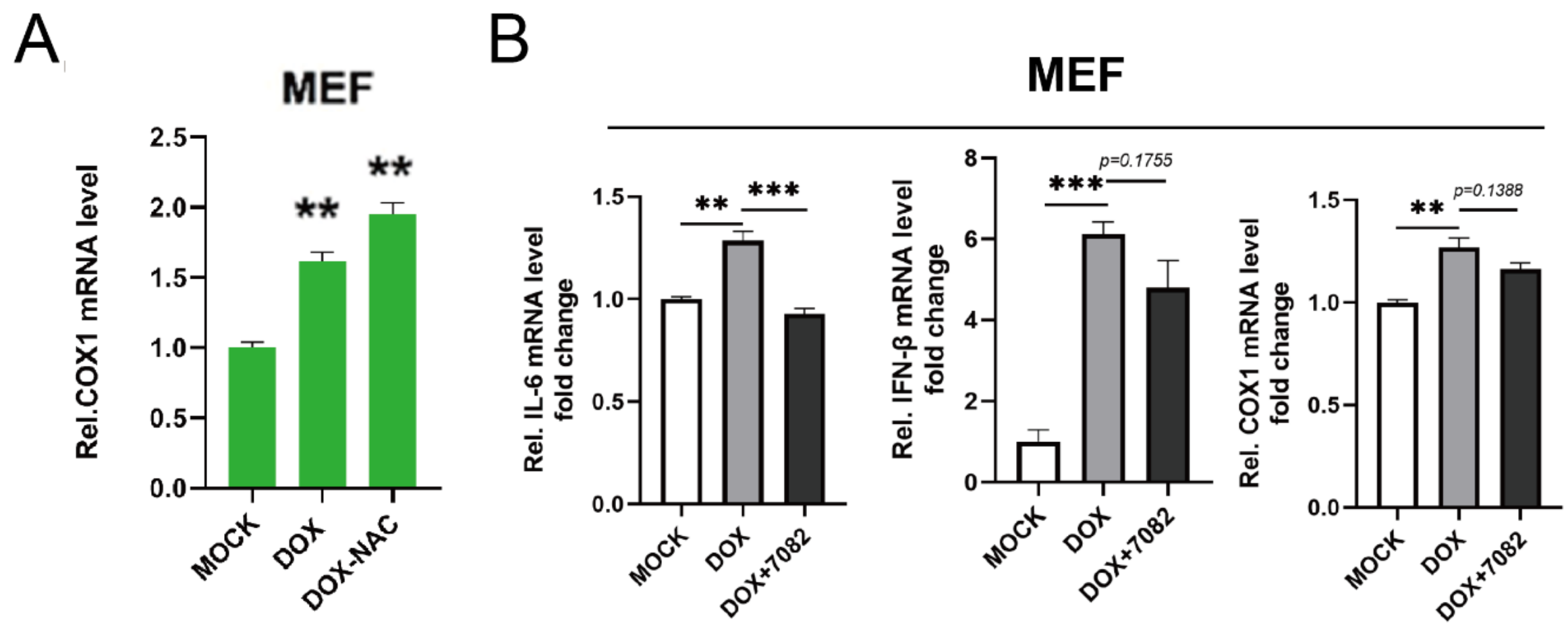

Supplementary Figure 1. (A, B) mtRNA levels in MEFs treated with DOX plus NAC (A) or BAY 11-7082 (B) were analyzed by qRT-PCR.

Data are presented as mean ± SEM and are representative of at least two independent experiments. Statistical significance was determined by Student's t-test; $^*P < 0.05$, $^{**}P < 0.01$, $^{***}P < 0.001$, $^{****}P < 0.0001$

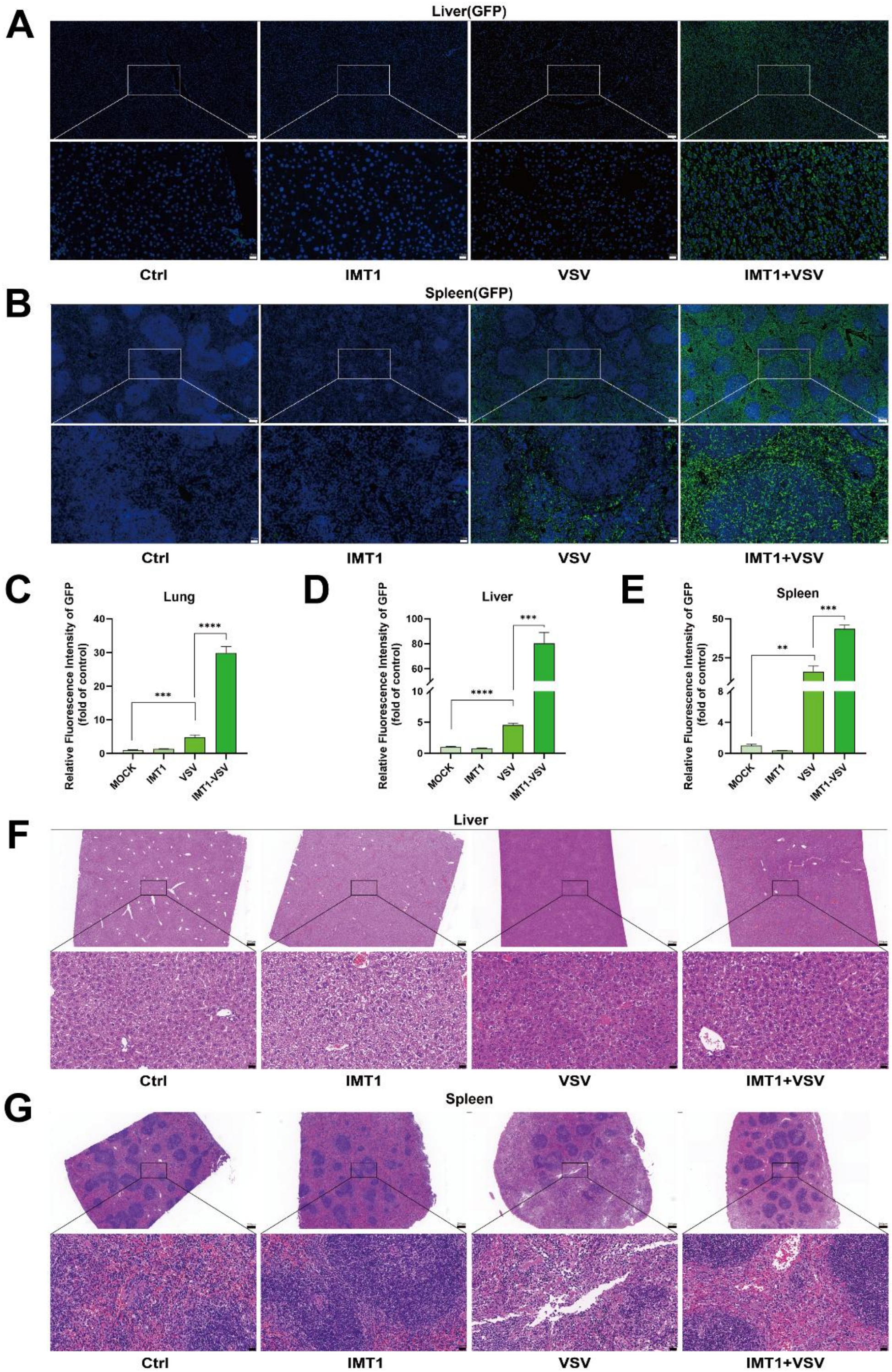

Supplementary Figure 2. (A-B) Immunofluorescence staining for GFP in Liver/Spleen tissues. (C-E) Quantitative analysis of GFP fluorescence in liver, lung, and spleen tissues. (F-G)H&E staining of Liver/Spleen tissues.

Data are presented as mean ± SEM and are representative of at least two independent experiments. Statistical significance was determined by Student's t-test; *$P < 0.05$, **$P < 0.01$, ***$P < 0.001$, ****$P < 0.0001$

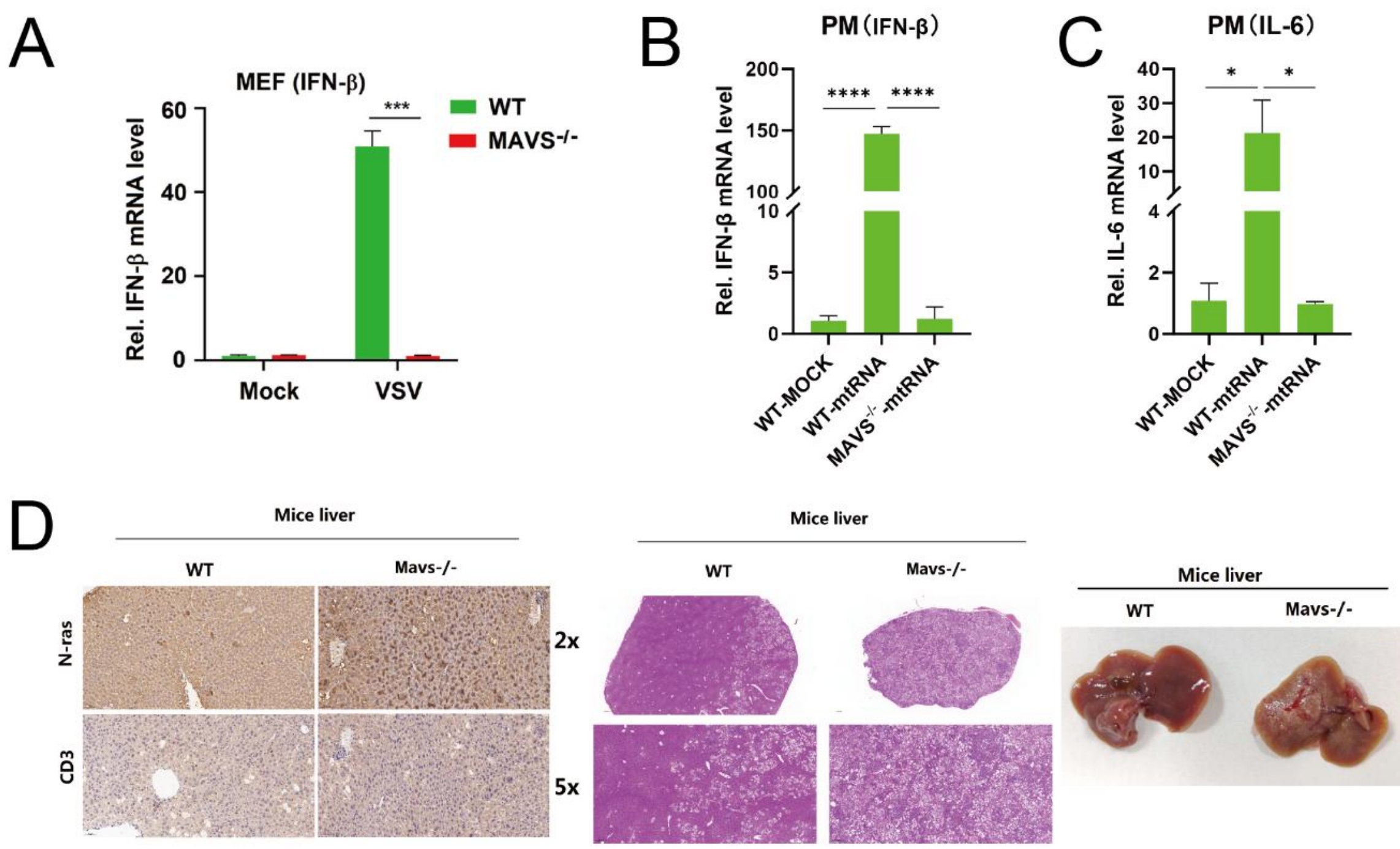

Supplementary Figure 3. (A) IFN-β mRNA levels in wild-type (WT) and MAVS-/- mouse embryonic fibroblasts (MEFs) infected with VSV (12h) were detected by qRT-PCR. (B, C) Purified mtRNA from donor peritoneal macrophages (PMs) was transfected into WT or MAVS-/- recipient PMs. (B) IFN-β and (C) IL-6 mRNA levels were measured. (D) WT and MAVS-/- mice underwent hydrodynamic injection of transposon-based vectors expressing myr-AKT and NRASV12. N-Ras and CD3+ T cell staining in liver at day 12. H&E staining of liver at 5 weeks post-injection. Representative liver morphology.

Data are presented as mean ± SEM and are representative of at least two independent experiments. Statistical significance was determined by Student's t-test; *P < 0.05, **P < 0.01, ***P < 0.001, ****P < 0.0001

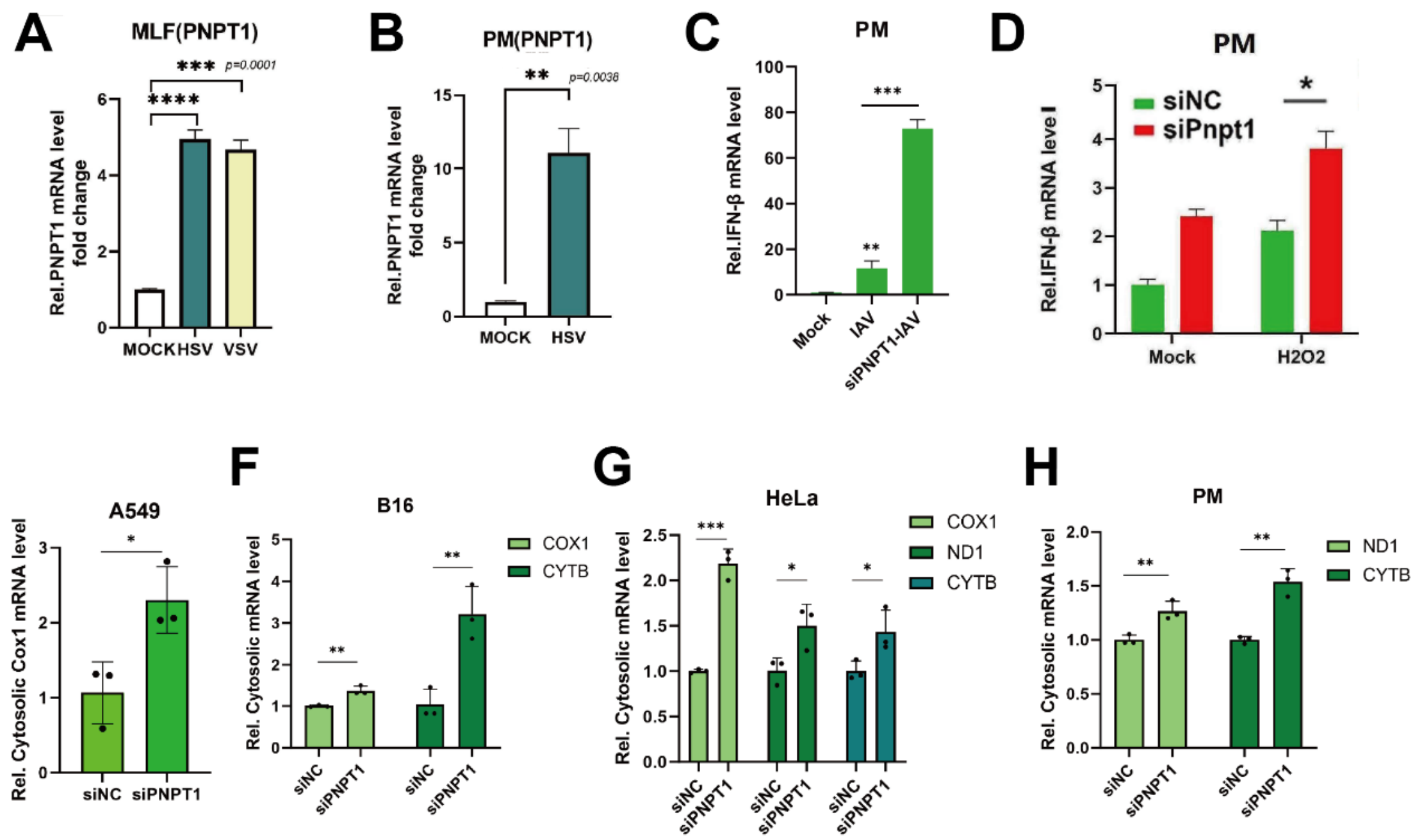

Supplementary Figure 4. (A-B) PNPT1 mRNA levels in mouse lung fibroblasts (MLFs) and peritoneal macrophages (PMs) infected with VSV (4 h) or HSV (4 h) were measured by qRT-PCR. (C) PMs infected with siPNPT1 (48 h) were challenged with VSV (12 h). IFN-β mRNA levels were quantified by qRT-PCR. (D) PMs transfected with siPNPT1 (48 h) were treated with H2O2 (6 h). IFN-β mRNA levels were analyzed by qRT-PCR. (E–H) Cytosolic COX1 mtRNA levels were measured in A549 (E), B16 (F), HeLa (G), and PM (H) cells after siPNPT1 treatment.

Data are presented as mean ± SEM and are representative of at least two independent experiments. Statistical significance was determined by Student's t-test; *P < 0.05, **P < 0.01, ***P < 0.001, ****P < 0.0001

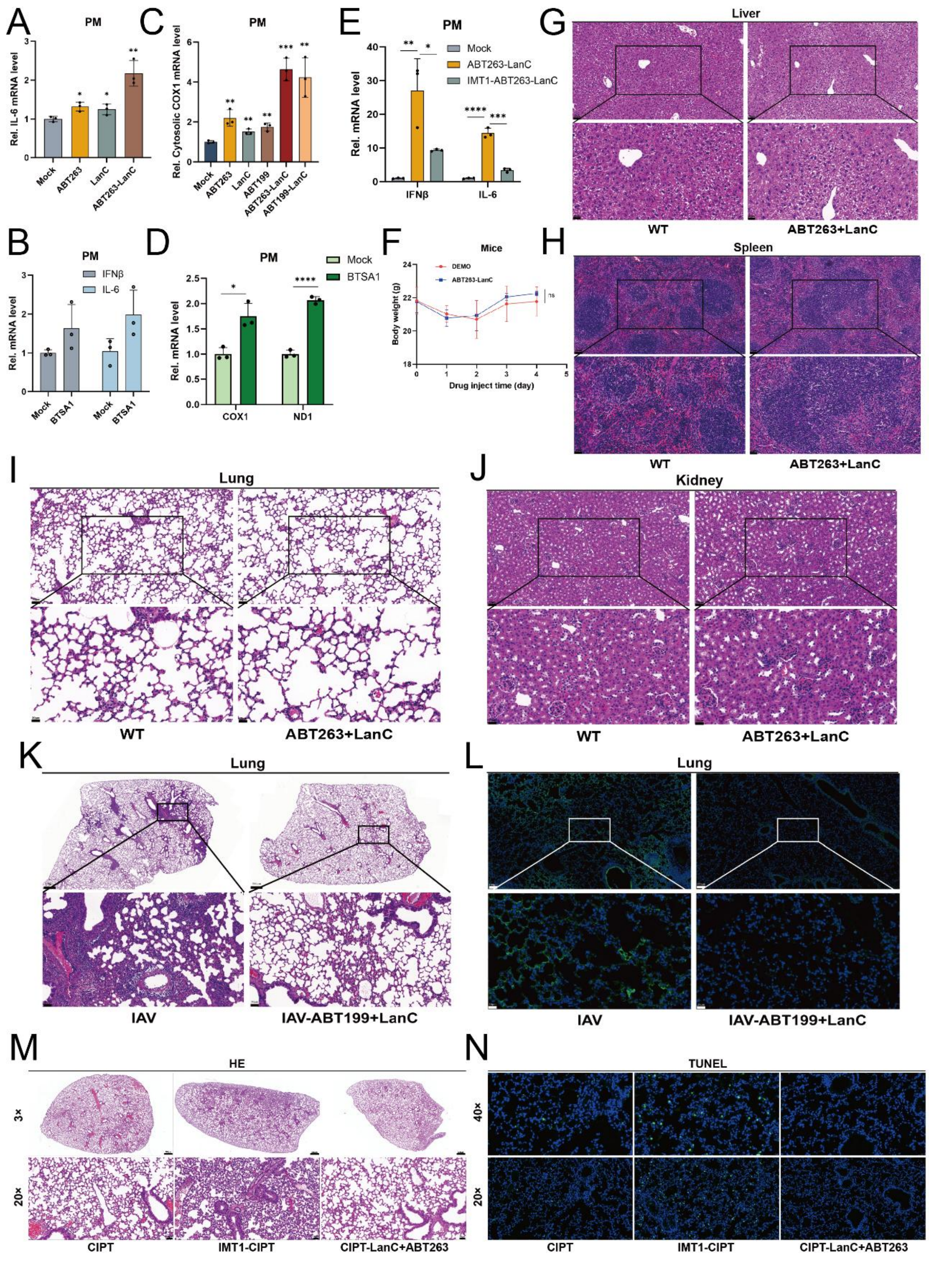

Supplementary Figure 5. (A-B) IL-6 or IFNβ mRNA levels in PMs treated with drug (6 h) were quantified by qRT-PCR. (C) Cytosolic mtRNA levels in PMs treated with drugs (6 h) were analyzed

by qRT-PCR. (D) mtRNA levels in PMs treated with drugs (6 h) were analyzed by qRT-PCR. (E) PMs were pretreated with IMT1 (2 h) followed by drugs stimulation (6 h). IL-6 or IFNβ RNA levels were analyzed by qRT-PCR. (F) Body weight changes in WT mice (8-week-old) following intraperitoneal injection of drugs (20 mg/kg). (G-H) Histological differences in liver (G), spleen (H), lung (I), and kidney (J) tissues between WT and combination drug treatment groups. (K) H&E staining of lung. (L) Immunofluorescence staining of viral nucleoprotein (NP). (M) H&E staining shows histopathological changes in lung sections from AKI mice treated with IMT1 or LanC+ABT263 following CIPT induction (25 mg/kg). (N) TUNEL staining shows apoptotic cells (green) in lung tissue from the same experimental groups.

Data are presented as mean ± SEM and are representative of at least two independent experiments. Statistical significance was determined by Student's t-test; $*P < 0.05$, $**P < 0.01$, $***P < 0.001$, $****P < 0.0001$
