# Supplementary material for: Mitochondrial RNA as a broad-spectrum DAMP coordinates antiviral and damage-related immune clearance and confers therapeutic potential
Source: Cell Death Dis. 2026 Aug 4;17(1):928. doi: 10.1038/s41419-026-09153-7 (PMC13724680; doi:10.1038/s41419-026-09153-7)
Supplement: Supplementary file 2 [file 41419_2026_9153_MOESM3_ESM.pdf]

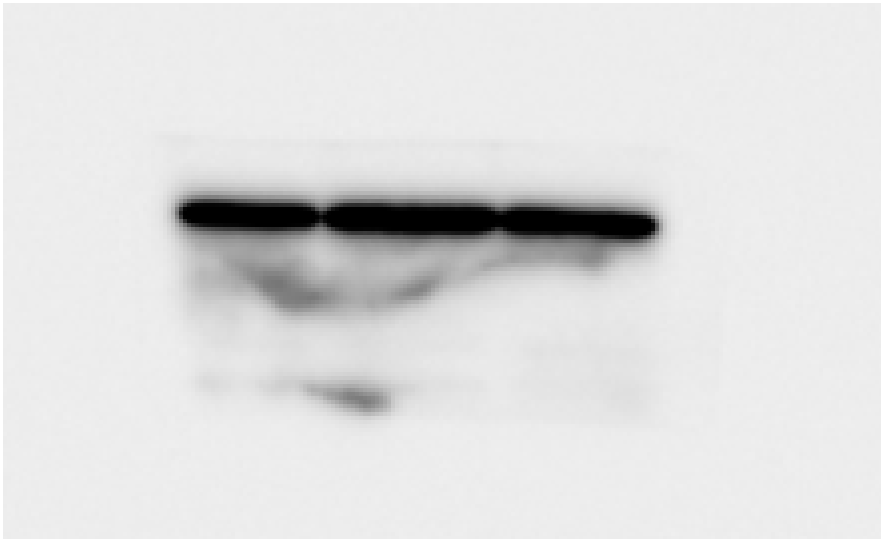
GAPDH（图 F）

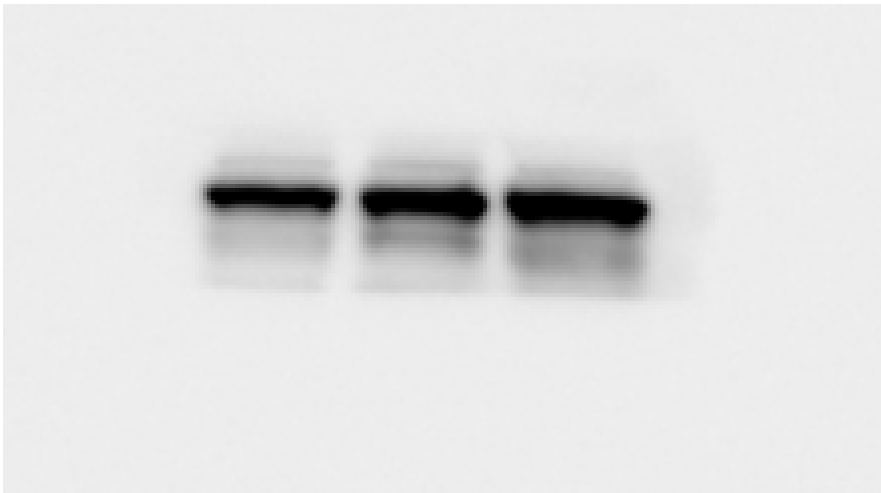
PNPT1（图 F）

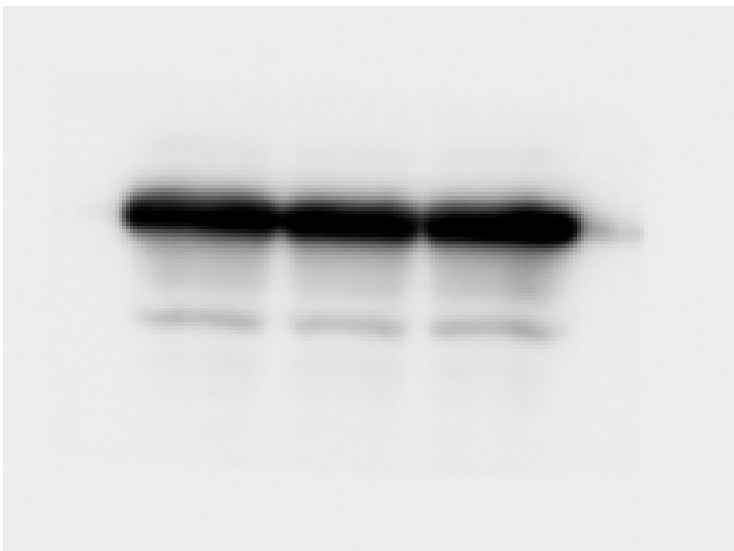
GAPDH（图 H）

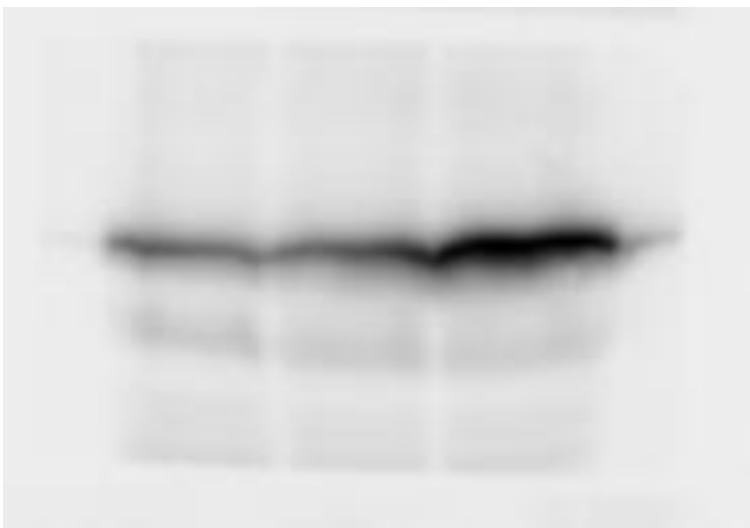
PNPT1（图 H）

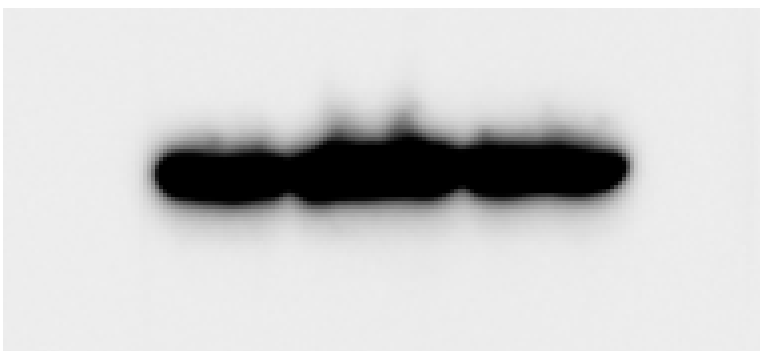
GAPDH（图 I）

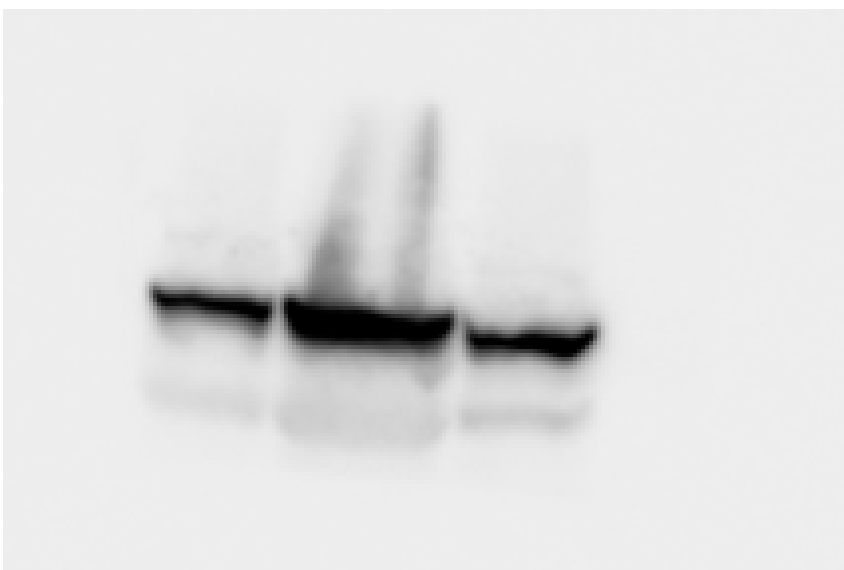
PNPT1(图 I)
