# Supplementary material for: Mitochondrial RNA as a broad-spectrum DAMP coordinates antiviral and damage-related immune clearance and confers therapeutic potential
Source: Cell Death Dis. 2026 Aug 4;17(1):928. doi: 10.1038/s41419-026-09153-7 (PMC13724680; doi:10.1038/s41419-026-09153-7)
Supplement: Supplementary file 3 [file 41419_2026_9153_MOESM4_ESM.docx]

| **Primer name** | **Reverse primer (5′→3′)** | **Forward primer (5′→3′)** |
| --- | --- | --- |
| GAPDH-Mouse | GGGGTCGTTGATGGCAACA | AGGTCGGTGTGAACGGATTTG |
| IL-6-Mouse | AATTAAGCCTCCGACTTGTGAA | TTCCATCCAGTTGCCTTCTTG |
| IFN-β-Mouse | TCAGAAACACTGTCTGCTGG | AGATCAACCTCACCTACAGG |
| CYTB-Mouse | GTACTGAATCCTAGTAGCCAA | AGTATGAGATGGAGGCTAGT |
| COX1-Mouse | ATAGGTTGGTTCCTCGAATG | CCTTTGCTTCAAAACGAGAA |
| ND1-Mouse | CGCCCTAACAACTATTATCTTCC | GAAGCGTGGATAAGATGCTC |
| ISG15-Human | CTCAGCCAGAACTGGTCTCTGT | CTCAGCCAGAACTGGTCTTCGT |
| IAV | TGCTCATTTTGATGGTGATAACCG | ATACGACTAGCAAAAGCAGGG |
| PNPT1-Human | GAGAGGGCTACGGAAGCGCCA | ACCGCTCTTTCCAGCTGGCTACT |
| IL-6-Human | CCATCTTTGGAAGGTTCAGGTTG | ACTCACCTCTTCAGAACGAATTG |
| IFN-β-Human | TGACTATGGTCCAGGCACAG | TTGTTGAGAACCTCCTGGCT |
| CYTB-Human | GGTGATTCCTAGGGGGTTGT | AGACAGTCCCACCCTCACAC |
| COX1-Human | TGGCGTAGGTTTGGTCTAGG | ACGTTGTAGCCCACTTCCAC |
| PNPT1-Mouse | CTCTGTCAGAGGAGCCAATCTC | GCCTTCCCAATTCATGCCGTTG |

**Table S1. Primer sequences used for quantitative real-time PCR.**
